# Effects of the COVID-19 pandemic on HIV service delivery and viral suppression: Findings from the SHARP program in Northern Nigeria

**DOI:** 10.1371/journal.pone.0300335

**Published:** 2024-04-02

**Authors:** Cyrus Mugo, Oluwasanmi Adedokun, Oluwafemi David Alo, Nnenna Ezeokafor, Sylvester Adeyemi, Zipporah Kpamor, Leila Madueke, Ezekiel James, Sylvia Bolanle Adebajo, Bazghina-werq Semo

**Affiliations:** 1 Kenyatta National Hospital, Nairobi, Kenya; 2 Center for International Health, Education, and Biosecurity (CIHEB), Maryland Global Initiatives Corporation (MGIC) – University of Maryland, Abuja, Nigeria; 3 Chemonics International, Abuja, Nigeria; 4 United States Agency for International Development, Abuja, Nigeria; 5 Chemonics International, Washington, D.C., United States of America; North Carolina State University, UNITED STATES

## Abstract

During the COVID-19 pandemic, HIV programs scaled up differentiated service delivery (DSD) models for people living with HIV (PLHIV). We evaluated the effects of COVID-19 on HIV service delivery and viral suppression in facilities in Northern Nigeria, and determined factors associated with viral suppression among adolescents and adults. We analysed a cross-sectional survey data from facility heads, and retrospective, routinely collected patient data from 63 facilities for PLHIV ≥10 years old in care between April 2019-March 2021, defining study periods as “pre-COVID-19” (before April 2020) and “during COVID-19” (after April 2020). For the pre-COVID and the COVID-19 periods we compared uptake of antiretroviral therapy (ART) refills of ≥3 months (MMD3), and ≥6 months (MM6), missed appointments, viral load (VL) testing, VL testing turnaround time (TAT) and viral suppression among those on ART for ≥6 months using two proportions Z-test and t-tests. We fit a multivariable logistic regression model to determine factors associated with maintaining or achieving viral suppression. Of 84,776 patients, 58% were <40 years, 67% were female, 55% on ART for >5 years, 93% from facilities with community-based ART refill, a higher proportion were on MMD3 (95% versus 74%, p<0.001) and MMD6 (56% versus 22%, p<0.001) during COVID-19 than pre-COVID-19, and a higher proportion had VL testing during COVID-19 (55,271/69,630, [84%]) than pre-COVID-19 (47,747/68,934, [73%], p<0.001). Viral suppression was higher during COVID-19 pandemic compared to the pre-COVID era (93% [51,196/55,216] versus 91% [43,336/47,728], p<0.001), and there was a higher proportion of missed visits (40% [28,923/72,359] versus 39% [26,304/67,365], p<0.001) and increased VL TAT (mean number of days: 38 versus 36, p<0.001) during COVID-19 pandemic and pre-COVID period respectively. Factors associated with maintaining or achieving suppression during COVID-19 were receiving MMD3 and MMD6 refills (OR: 2.8 [95% CI: 2.30–3.47] and OR: 6.3 [95% CI: 5.11–7.69], respectively) and attending clinics with community-based ART refill (OR: 1.6 [95% CI: 1.39–1.87]). The program in Northern Nigeria demonstrated resilience during the COVID-19 pandemic and adoption of MMD had a positive impact on HIV care. Though VL TAT and missed clinic visits slightly increased during the pandemic, VL testing improved and viral suppression moved closer to 95%. Adoption of MMD and community-based models of care at scale are recommended for future pandemic preparedness.

## Introduction

In 2020, the coronavirus disease 2019 (COVID-19) [1, 2] caused by SARS-COV-2 virus [1] was declared a global pandemic [2]. The primary identified mode of transmission of the SARS-COV-2 virus is through exposure to respiratory droplets and aerosols when infected persons sneeze, cough or talk [1]. Globally, there were over half a billion reported cases of COVID-19 and over 6 million deaths reported [3], with Nigeria accounting for over 250,000 cases and more than 3,000 deaths [4]. The COVID-19 pandemic thus put additional strain on already burdened health systems in low- and middle-income countries, and reduced access to other essential health services [5]. Several countries with high burdens of HIV scaled up differentiated models of care for people living with HIV (PLHIV) [6, 7] to mitigate against the impact of the COVID-19 movement restrictions, including: community-based HIV case finding (specifically targeted community testing and index case testing); community-based distribution of antiretroviral therapy (ART); multi-month dispensing of ART (MMD) [8, 9]; and community-based viral load (VL) sample collection. In Nigeria, community-based ART distribution models included: community ART groups (patients take turns collecting and distributing treatment from health facilities on behalf of PLHIV group members) [10, 11]; family drug pick-up groups (family members take turns collecting and distributing treatment from health facilities on behalf of other family members); community pharmacy refill programs (patients pick up their ART at designated community pharmacies [10, 11]); and decentralized drug distribution centers (lay providers dispense ART at designated locations within the community [10]). These community-based ART distribution models are now widely accepted in many countries in sub-Saharan Africa [12]. For example, in a study of 21 PEPFAR-supported countries, MMD coverage grew from 49% to 74% between December 2019 (pre-COVID-19) and September 2020 (during COVID-19), respectively [7]. Specifically, in Nigeria, MMD coverage grew from 53% to 94% within the same period [13]. This rapid growth in MMD may be attributable to the expansion of these services to PLHIVs not defined as “stable” (i.e., those on ART for at least one year with no adverse reactions or current illness; not pregnant or breastfeeding; with good understanding of lifelong ART adherence; and at least two consecutive VL measurements of less than 1000copies/mm [14, 15]. Few studies have evaluated system efficiency and patient outcomes related to HIV care during the COVID-19 pandemic. Some studies have shown a decrease in viral load testing during COVID-19 compared to pre-COVID-19 period [16–18]. Viral load turnaround time was also observed to have increased between pre-COVID-19 and during COVID-19 period in a study in North East Nigeria [19]. Furthermore, a systematic review of data from five studies from two high income countries (USA and Italy) consistently showed no significant difference in viral suppression between pre-COVID-19 and COVID-19 period [20]. Similar findings were observed from studies in Malawi [17], South Africa [18], and the United States [16]. Appointment keeping was however observed to have reduced between pre-COVID-19 and COVID-19 period in an Ethiopian study [19]. To the best of our knowledge, no single study has evaluated the effect of COVID-19 pandemic on all these outcomes in Nigeria. This study, therefore, evaluated the effects of COVID-19 on the uptake of MMD, VL testing, VL turnaround time, viral suppression, and missed appointments in health facilities; and assessed factors associated with viral suppression among PLHIV ages ≥ 10 years.

## Methods

We conducted retrospective analyses of routinely collected longitudinal program data for PLHIV 10 years of age and older in Northern Nigeria, supplemented with a cross-sectional survey for facility-level data completed by heads of the HIV clinics in the facilities.

### Study setting and context

#### Facility survey

Heads of HIV clinics for all facilities supported by the Nigeria Strategic HIV/AIDS and Tuberculosis (TB) Response Program (SHARP) excluding 4 facilities which were inaccessible for security reasons, were recruited to complete the interviewer-administered facility survey, which collected information on the adoption of differentiated service delivery models in the facility including community delivery models of ART. The clinics were located in 11 states in Northern Nigeria (Kebbi, Sokoto, Kano, Jigawa, Zamfara, Borno, Bauchi, Adamawa, Yobe, Niger, and Kwara) in which HIV prevalence ranged between 0.3–1.6% in 2018 [21]. All HIV clinics had >300 clients and were a mix of university teaching hospitals, public and faith-based private facilities, with approximately 20 of the 63 located in rural areas.

#### Routine patient data

Data were abstracted from 63 HIV clinics in Northern Nigeria supported by the SHARP program. All facilities used the Lafiya Management Information System (LAMIS) electronic medical records systems (EMR) for patient management [9].

### Data collection and management

We abstracted data on patient demographics, clinical encounters, ART refills and VL testing from the LAMIS databases at the facilities for the period between April 1, 2019, and March 31, 2021. Patient personal identifiable information (names, addresses and telephone numbers) were replaced with system-generated unique patient identifiers during the export process and imported to Stata software for analyses. Data abstraction was conducted between August 15-October 31, 2021.

Data on facility characteristics and community ART refill models were collected using a facility survey completed by 63 heads of HIV clinics or their designates. The questionnaire was developed in an electronic format using Open Data Kit (ODK) and administered using android tablet devices. We merged survey data with routine data using the unique facility ID to create one analytic dataset.

### Study measures

The main outcome variables for this study were: MMD3 and MMD6 utilization, VL testing; VL turn-around time, viral suppression; and missed clinical appointments. The study period was divided into “pre-COVID-19” (April 1, 2019 –March 31, 2020) and “during COVID-19” (April 1, 2020 –March 31, 2021) phases. VL testing was assessed among patients on ART for at least 6 months. Viral suppression was assessed among those with at least one VL result and defined as having <1000 copies/ml. For patients with multiple VL results in the pre-COVID-19 or during COVID-19 periods, we used the latest VL result for that period. A refill appointment was defined as “missed” if there was no record of a visit more than 28 days after a scheduled date. Other outcome variables included VL turn-around-time (TAT), defined as duration between the sample collection date and the date the VL result was received at the clinic. MMD model types were defined as “MMD3” if ART refill return dates were at least 3 months later; we further defined a sub-set with ART refill dates of at least 6 months later at any visit as “MMD6.” Facilities were considered to offer community-based ART refill services if they had at least one of the following: community ART groups: family drug pick up groups: community pharmacy refill: and decentralized drug distribution centers.

Independent variables considered in the analyses include sex categorized as male or female; age categorized in 10-year age bands: 10–19 years, 20–29 years, 30–39 years, 40–49 years and ≥ 50 years. Regions were categorized as North-West, North Central and North-East. Duration on ART was categorized as 0–5 years, 6–10 years, and ≥ 11 years. Facility types were categorized as with- or without- community models.

### Data analyses

Frequencies and proportions were used to describe categorical variables while median and interquartile ranges were computed for numerical variables. Bivariate analyses using two proportions Z-test were used to examine the differences in proportion of MMD uptake, VL testing, viral suppression and missed refill appointments pre- and during COVID-19. Differences in the average pre- and during COVID-19 VL TAT were examined using t-tests.

We estimated the impact of the COVID-19 pandemic on viral suppression for persons who were in care before and during COVID-19. Change in viral suppression was assessed among participants who had at least one VL result in both COVID-19 periods. Participants were assigned “positive change” in viral suppression if they were: 1) virally suppressed at both COVID-19 periods, or 2) unsuppressed pre-COVID-19 but suppressed during the COVID-19 period. They were assigned “negative change” if: 1) the VL result during the COVID-19 period was unsuppressed, or 2) both pre- and during COVID-19 periods were unsuppressed. A multivariable logistic regression model was fitted to determine factors associated with a positive change in viral suppression (maintaining or achieving viral suppression during the COVID-19 period). Variables selected included participant: sex; age group; region; duration on ART; maximum ART refill (<3 months, 3–5 months, and ≥6 months); and facility classification. We reported adjusted odds ratios (aOR) and 95% confidence intervals (95%CI). P-values ≤ 0.05 were considered statistically significant for all analyses. All analyses were done using Stata 16 (StataCorp, College Station, TX).

### Ethical considerations

This study was approved by the National Health Research Ethics Committee of Nigeria (NHREC/01/01/2007-28/06/2021B) and the Institutional Review Board of the University of Maryland, Baltimore (HP-00097414). Individual consent for the abstraction of patient medical records was waived. The analyses used de-identified data and those who performed the data extraction and collection were not involved in the data analyses or included as co-authors. Written informed consent was provided by the heads of facilities or designates who completed the facility survey.

## Results

Of 101,320 patients’ who had VL, and pharmacy records abstracted, 16,544 (16%) were excluded from the analyses because of incomplete data. Of the 84,776 patients included in the analyses, median age was 37 years (interquartile range: 31–45 years), 67% were female, and 55% had been on ART for more than 5 years. Approximately 93% were receiving treatment in health facilities that had community-based models of ART refill (Table 1).

**Table 1:** Characteristics of study participants.

Characteristics (N = 84,776)	n (%)
**Sex**	
Female	57,044 (67)
Male	27,732 (33)
**Age group**	
10–19 years	3,508 (4)
20–29 years	14,914 (18)
30–39 years	30,250 (36)
40–49 years	22,427 (26)
50+ years	13,677 (16)
**Regions**	
North-Central	14,714 (17)
North-East	35,577 (42)
North-West	34,485 (41)
**Duration on ART**	
0–5 years	37,889 (45)
6–10 years	29,707 (35)
11+ years	17,175 (20)
**Facility type**	
Without community models	5,899 (7)
With community-based models	78,877 (93)

### Uptake of MMD

**Fig 1:**
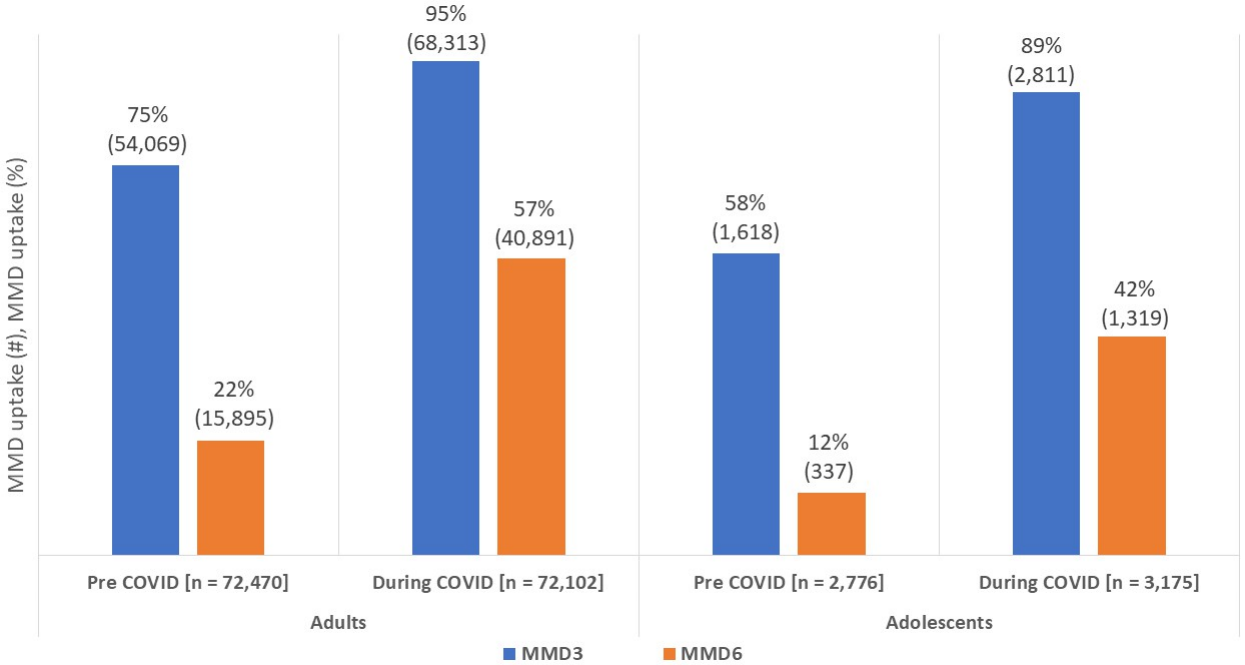
Multi-month dispensing before and during the COVID-19 pandemic.

There were significant differences in the proportion of patients on MMD3 models in the pre-COVID-19 period (74% [55,687/75,246]) and during COVID-19 (95% [71,124/75,277]), p<0.001). The increase in MMD3 utilization was highest among adolescents (10–19 years), from 58% to 89%; while for adults (>19 years), utilization increased from 75% to 95%. The utilization of MMD6 was significantly higher during the COVID-19 period (22% [16,232/75,246] versus 56% [42,210/75,277], p<0.001). The increase in adoption of MMD6 was similar among adolescents (12% to 42%) as adults (22% to 57%) (Fig 1).

### Differences in VL testing, viral suppression and missed visits pre- and during the COVID-19 pandemic

Among the 68,934 patients in care for >6 months in the pre-COVID-19 period, 47,747 (69%) had a VL test. This was significantly lower than the 55,271/69,630 (79%) who received a VL test during the COVID-19 pandemic (p<0.001). There was a slight increase in viral suppression among those tested in the COVID-19 period (93% [51,196/55,216]) compared to the pre-COVID-19 period (91% [43,336/47,728], p<0.001). In the pre-COVID-19 period, 39% (26,304/67,365) of patients had at least one missed refill visit. This increased during the COVID-19 pandemic, with 40% (28,923/72,359) of patients having at least one missed refill visit (p<0.001) (Table 2). The VL TAT also increased during the pandemic, with mean number of days pre-COVID-19 being 36.6 (95%CI: 36.4–36.9) and during the pandemic being 38.1 (95%CI: 37.8–38.3, p<0.001).

**Table 2:** Viral load testing, viral suppression and missed appointments pre- and during COVID.

Variables	Pre-COVID		During COVID		P-value
	N	n (%)	N	n (%)	
VL test done	68,934	47,747 (69.3)	69,630	55,271 (79.4)	<0.001
VL suppression	47,728	43,336 (90.8)	55,216	51,196 (92.7)	<0.001
Missed appointment	67,365	26,304 (39.1)	72,359	28,923 (40.0)	<0.001

### Factors associated with a positive change in viral suppression during the COVID-19 pandemic

We found evidence of an association between the following factors and a positive change in viral suppression: sex; age; MMD3; MMD6; facilities with community-based ART refill models; and the region where a patient resided. Compared to female patients, male patients were less likely to have a positive change in viral suppression (aOR: 0.80 [95%CI: 0.75–0.90]). Compared to adolescents (<20 years), persons in the older age groups had higher odds of a positive change in viral suppression (from aOR: 2.6 [95%CI: 2.21–3.15] for those in the 20–29 years age group, to aOR: 4.9 [95%CI: 4.11–5.96] for those in the >50 years age group). Patients who had ever received a 3–5 months ART refill and ≥6 months ART refill had higher odds of a positive change in viral suppression (aOR: 2.8 [95%CI: 2.30–3.47] and aOR: 6.3 [95%CI: 5.11–7.69], respectively) compared to patients who only received <3 months refills. Furthermore, patients who attended clinics in facilities with community-based models of ART refill had higher odds of a positive change in viral suppression (aOR: 1.6 [95%CI: 1.39–1.87]). Lastly, patients in the North-East and North-West regions were more likely to have a positive change in viral suppression compared to those in the North-Central region (aOR: 1.5 [95%CI: 1.36–1.72] and aOR: 1.3 [95%CI: 1.18–1.49]) (Table 3).

**Table 3:** Factors associated with a positive change in viral suppression over COVID-19 periods.

Characteristics	Positive change in viral suppression		aOR (95%CI)	p-value
	N^α^	n (%)		
**Sex**				
Female	25,979	24,345 (93.7)	Reference	
Male	11,273	10,462 (92.8)	0.8 (0.75–0.90)	<0.001
**Age group**				
10–19 years	1,314	1,046 (79.6)	Reference	
20–29 years	5,363	4,945 (92.2)	2.6 (2.21–3.15)	<0.001
30–39 years	13,736	12,840 (93.5)	3.1 (2.67–3.65)	<0.001
40–49 years	10,483	9,895 (94.4)	3.7(3.17–4.39)	<0.001
50+ years	6,356	6,081 (95.7)	4.9 (4.11–5.96)	<0.001
**Regions**				
North-Central	6,214	5,599 (90.1)	Reference	
North-East	18,070	17,156 (94.9)	1.5 (1.36–1.72)	<0.001
North-West	12,968	12,052 (92.9)	1.3 (1.18–1.49)	<0.001
**Duration on ART**				
0–5 years	12,634	11,671 (92.4)	Reference	
6–10 years	15,947	14,933 (93.6)	1.1 (0.99–1.19)	0.086
11+ years	8,671	8,203 (94.6)	1.1 (0.99–1.26)	0.083
**Maximum ART refilled**				
<3 months	584	437 (74.8)	Reference	
3–5 months	10,386	9,266 (89.2)	2.8 (2.30–3.47)	<0.001
≥6 months	26,282	25,104 (95.5)	6.3 (5.11–7.69)	<0.001
**Facility type**				
Without community models	2,333	2,104 (90.2)	Reference	
With community-based models	34,919	32,703 (93.6)	1.6 (1.39–1.87)	<0.001

^α^Patients with viral load results at pre- and during COVID-19

## Discussion

In this study, we sought to evaluate the effects of COVID-19 on the uptake of MMD, VL testing, VL turnaround time, viral suppression, and missed appointments in health facilities; and assessed factors associated with viral suppression among PLHIV ages ≥ 10 years. In the COVID-19 period, more patients (adolescents and adults) were introduced to MMD of ART compared to the pre-COVID-19 period. The proportions of eligible patients who received a VL test and were virally suppressed in the first year of the COVID-19 pandemic were also higher than in the year preceding the pandemic. However, this study also established that a slightly higher proportion of patients missed their appointments and that the TAT from sample collection to receiving VL results also significantly increased during the COVID-19 period. Factors associated with a positive change in viral suppression during the COVID-19 period were, being female; older age; utilizing a MMD model; and clinic attendance at a facility with community based-models.

Our study findings of increased MMD uptake during the COVID-19 period are consistent with other studies in Nigeria and the rest of sub-Saharan Africa [7, 8]. Since 2016, PEPFAR had been promoting MMD uptake as a strategy for improved patient treatment retention [22]. The strategy aims to reduce the frequency of visits to health facilities for routine ART drug refill as a way of decreasing life disruptions for patients and improving their adherence and retention on treatment, as well as lessening healthcare workers’ service delivery burden [22, 23]. During the COVID-19 period, the rollout of MMD received additional agency as a measure to reduce patient visits to the health facility, thereby decreasing their exposure to COVID-19 transmission [24].

Counterintuitively, we observed an increase in VL testing during COVID-19 compared to the pre-COVID-19 period, a finding that is in contrast to that of Atuhaire et al., in Uganda [25]. The increase in our study could be attributed to specific interventions by the SHARP program in Northern Nigeria, such as: establishing VL sample collection centers within the communities (thus bringing the service closer to patients and creating an incentive to improve uptake of testing); and sending appointment reminders via text messaging [24]. Notably, this improvement was replicated in the national program, through which VL testing coverage increased from 80% as of December 2019 [26] to 88% by December 2020 [13]. This may be attributable to efforts of programs like SHARP that implemented the PEPFAR guidance [6].

The improvement in viral suppression during the pandemic is a continuation of trends over the last few years. While it was expected that the COVID-19 pandemic would negatively impact treatment outcomes, the positive effects of MMD, community-based ART refill and improved VL testing, all of which received more support and opportunity during the pandemic, were key mitigating interventions. Previous studies, for example, Bailey et al., also demonstrated higher viral suppression during the COVID-19 pandemic partly due to the rollout of MMD [7]. Further, since 2018, the preferred first line ART regimen in Nigeria has been tenofovir/lamivudine/dolutegravir (TLD) [27]. The COVID-19 period coincided with the peak of the dolutegravir rollout in Nigeria [26]. Patients on the dolutegravir containing regimen have been shown to achieve and maintain higher rates of viral suppression when compared to other regimens [28–30], with some achieving viral suppression as early as four weeks after taking the drug [31]. We found differences in viral suppression across regions, with the North-Central region having poorer viral suppression. This finding is similar to the report by Tomescu et al., [32] who found higher odds of viral non-suppression in the North-Central zone, likely related to poorer health seeking behaviors and adherence to treatment, linked to religious beliefs [32].

This study also demonstrated some negative effects of the COVID-19 pandemic. For instance, the TAT for processing samples for VL testing increased slightly during the COVID-19 period. Studies have reported varying impacts of the COVID-19 pandemic on laboratory TAT, with some conducted in high-income countries showing no differences between the COVID-19 and pre-COVID-19 periods [33]. On the other hand, in a South African study, there were initial increases in HIV VL testing TAT, which were later addressed through strategies such as having additional temporary staff at peak periods and process automations [34]. We postulate that increased uptake of VL testing for PLHIV during the COVID-19 period, as well as the additional burden of SARS-COV-2 testing at the molecular laboratories, may have contributed to the longer TAT. Expanding the molecular testing infrastructure and available human resource in the country will better position the country to handle future pandemics. Secondly, despite the successful adoption of MMD, there was a slight increase in the proportion of patients with at least one missed refill visit. A study in Ghana also reported initial declines in clinic attendance during the COVID-19 pandemic largely due to fear of contracting COVID-19 infection [35]. Within Nigeria, however, there is mixed evidence, with another study [8] showing improved patient retention and appointment keeping during the COVID-19 period. It is likely that patients missed their appointments when they had excess stock of medication accumulated from previous refills, a phenomenon described as stockpiling [36, 37]. The availability of community-based refills may also have contributed to this tendency, especially if the records were not updated to reflect the out-of-facility refills. In some instances, over-estimation of attrition from care has been associated with gaps in documentation [38].

Factors that were associated with maintaining or achieving viral suppression during the COVID-19 pandemic included: being female; older age; utilizing an MMD model: and attending HIV care at a facility with community based-models. These findings are consistent with previous studies showing that women living with HIV have better outcomes compared to men, especially viral suppression [39–42]. Older age has also previously been linked to better treatment outcomes, especially when comparing adults to adolescents [39, 43]. Other studies have also shown that PLHIV over 50 years of age have good rates of viral suppression [43]. In addition, as demonstrated in previous studies, MMD is associated with improved viral suppression [7], and patients receiving care using community-based models have higher odds of viral suppression [9]. Women have been known to have better health seeking behaviour [44, 45] and better adherence to treatment than men [46] which would consequently lead to improved viral suppression [47]. Similarly, adults have been shown to be more likely to be adherent to ART compared to adolescents and young persons [48–50], while MMD and community-based ART models are proven strategies for improving ART adherence [22, 23, 32]. Targeted program interventions to these affected socio-demographics may enhance viral suppression.

The Joint United Nations Program on HIV/AIDS has set the ambitious goal of ending HIV by 2030. To achieve this, 95% of all people living with HIV should know their HIV status, 95% of all people with diagnosed HIV infection should receive sustained antiretroviral therapy, and 95% of all people receiving antiretroviral therapy should be virally suppressed [51]. Achieving the third 95 is dependent upon, increased VL testing access and coverage, and quick turn-around of VL results to inform follow up actions. In addition, optimal appointment keeping is essential for retaining 95% of PLHIV on treatment, and ultimately achieving 95% viral suppression. Our study contributes to the knowledge gap, by providing evidence on the key metrics needed to inform program modifications towards achieving the UNAIDS second and third 95 goals in Nigeria.

A strength of our study included the large dataset from HIV clinics spread across three geopolitical zones in Nigeria, which enabled detection of differences in key outcomes between the COVID-19 periods, and assessment of factors associated with changes in viral suppression. However, the use of routine clinical data from EMR presented the challenge of missing data for many patients. Furthermore, at the time of this study, given that the EMR were not enabled to collect data on individual patients receiving care through community-based models, these data were analyzed at the facility level.

## Conclusion

These analyses demonstrate that despite the constraints imposed by the COVID-19 pandemic, the Northern Nigeria SHARP program was able to continue providing high-quality services with good clinical outcomes for patients. However, the COVID-19 pandemic resulted in longer TAT for VL tests and an increase in missed refill appointments. To lessen the effects of COVID-19 and any future pandemics, we recommend that programs adopt MMD and community-based models of care. Future studies should explore the effects on outcomes of the various models of community-based care accessed by patients.

## Supporting information

S1 File Inclusivity in global research. (DOCX)
